# Sex-Biased Evolutionary Forces Shape Genomic Patterns of Human Diversity

**DOI:** 10.1371/journal.pgen.1000202

**Published:** 2008-09-26

**Authors:** Michael F. Hammer, Fernando L. Mendez, Murray P. Cox, August E. Woerner, Jeffrey D. Wall

**Affiliations:** 1 ARL Division of Biotechnology, University of Arizona, Tucson, Arizona, United States of America; 2 Department of Ecology and Evolutionary Biology, University of Arizona, Tucson, Arizona, United States of America; 3 Institute for Human Genetics, University of California San Francisco, San Francisco, California, United States of America; Stanford University, United States of America

## Abstract

Comparisons of levels of variability on the autosomes and X chromosome can be used to test hypotheses about factors influencing patterns of genomic variation. While a tremendous amount of nucleotide sequence data from across the genome is now available for multiple human populations, there has been no systematic effort to examine relative levels of neutral polymorphism on the X chromosome versus autosomes. We analyzed ∼210 kb of DNA sequencing data representing 40 independent noncoding regions on the autosomes and X chromosome from each of 90 humans from six geographically diverse populations. We correct for differences in mutation rates between males and females by considering the ratio of within-human diversity to human-orangutan divergence. We find that relative levels of genetic variation are higher than expected on the X chromosome in all six human populations. We test a number of alternative hypotheses to explain the excess polymorphism on the X chromosome, including models of background selection, changes in population size, and sex-specific migration in a structured population. While each of these processes may have a small effect on the relative ratio of X-linked to autosomal diversity, our results point to a systematic difference between the sexes in the variance in reproductive success; namely, the widespread effects of polygyny in human populations. We conclude that factors leading to a lower male versus female effective population size must be considered as important demographic variables in efforts to construct models of human demographic history and for understanding the forces shaping patterns of human genomic variability.

## Author Summary

Like many primate species, the mating system of humans is considered to be moderately polygynous (i.e., males exhibit a higher variance in reproductive success than females). As a consequence, males are expected to have a lower effective population size (*N_e_*) than females, and the proportion of neutral genetic variation on the X chromosome (relative to the autosomes) should be higher than expected under the assumption of strict neutrality and an equal breeding sex ratio. We test for the effects of polygyny by measuring levels of neutral polymorphism at 40 independent loci on the X chromosome and autosomes in six human populations. To correct for mutation rate heterogeneity among loci, we divide our diversity estimates within human populations by divergence with orangutan at each locus. Consistent with expectations under a model of polygyny, we find elevated levels of X-linked versus autosomal diversity. While it is possible that multiple demographic processes may contribute to the observed patterns of genomic diversity (i.e., background selection, changes in population size, and sex-specific migration), we conclude that an historical excess of breeding females over the number of breeding males can by itself explain most of the observed increase in effective population size of the X chromosome.

## Introduction

Many studies have demonstrated large differences between males and females in the forces of evolution, i.e., mutation, recombination, selection, gene flow, and genetic drift. For example, mutation rates are often higher in males while females tend to have higher rates of recombination [1]. While the effects of sex-biased mutation and recombination have been directly estimated through genetic studies, we know very little about the extent to which sex-specific differences in gene flow and genetic drift have shaped patterns of variation at the level of the genome. For mammals, it is well known that females and males do not exhibit symmetrical behavior with respect to mating and dispersal practices. For instance, the typical mammalian system is characterized by polygyny (a mating practice in which a minority of males sire offspring with multiple females) and female philopatry (the tendency for females to breed at or near their place of origin) [2]. The development of sex-specific markers in humans has been instrumental in providing insights into the effects of sex-specific demographic processes. Contrasting patterns of diversity on the mitochondrial DNA (mtDNA) and non-recombining portion of the Y chromosome (NRY) have been interpreted to reflect sex-specificity in the rate and scale of migration and in effective population size [3]–[5]. However, these patterns could also reflect different molecular properties of these two haploid systems, differential selection, or stochasticity in the evolutionary process [5]. Unlike mtDNA and the NRY, the autosomes and X chromosome undergo recombination and contain numerous evolutionarily independent loci. Additionally, selection only affects those loci that are closely linked to selected sites. Consequently, different patterns of neutral polymorphism associated with the X chromosome and autosomes may be more directly ascribed to demographic differences between females and males.

Under standard models of DNA sequence evolution [6], the level of neutral polymorphism expected at equilibrium is governed by the product of *N_e_* (the effective population size) and the mutation rate. Since males carry only one X chromosome, the ratio of the X chromosome effective population size (*N_x_*) to the autosomal effective population size (*N_a_*) is expected to be ∼0.75 in simple models of a randomly mating population with equal numbers of breeding males and females (i.e., neutral models). Equivalently, if we correct for any differences in mutation rates across chromosomes, the X chromosome should have roughly 75% of the genetic diversity of the autosomes. However, under more complicated models the ratio of X to autosomal diversity levels can vary considerably [7]. For example, in populations with a female-biased sex-ratio, X-linked diversity will be higher than 75% of autosomal diversity [8], while in populations that have undergone recent population bottlenecks X-linked diversity will generally be less than 75% of autosomal diversity [9],[10]. In addition, if directional selection typically operates on mutations that are at least partly recessive, standard theory predicts that levels of diversity at linked neutral sites will be differentially affected depending on the chromosomal mode of inheritance. For advantageous recessive mutations, hemizygosity in males leads to a higher fixation rate on the X chromosome relative to the autosomes. This in turn will lead to less variability on the X chromosome relative to the autosomes due to the increased prevalence of genetic ‘hitchhiking’ [11]–[13]. In contrast, widespread purifying or background selection should reduce diversity on the autosomes more so than on the X chromosome [11].

**Figure 1:**
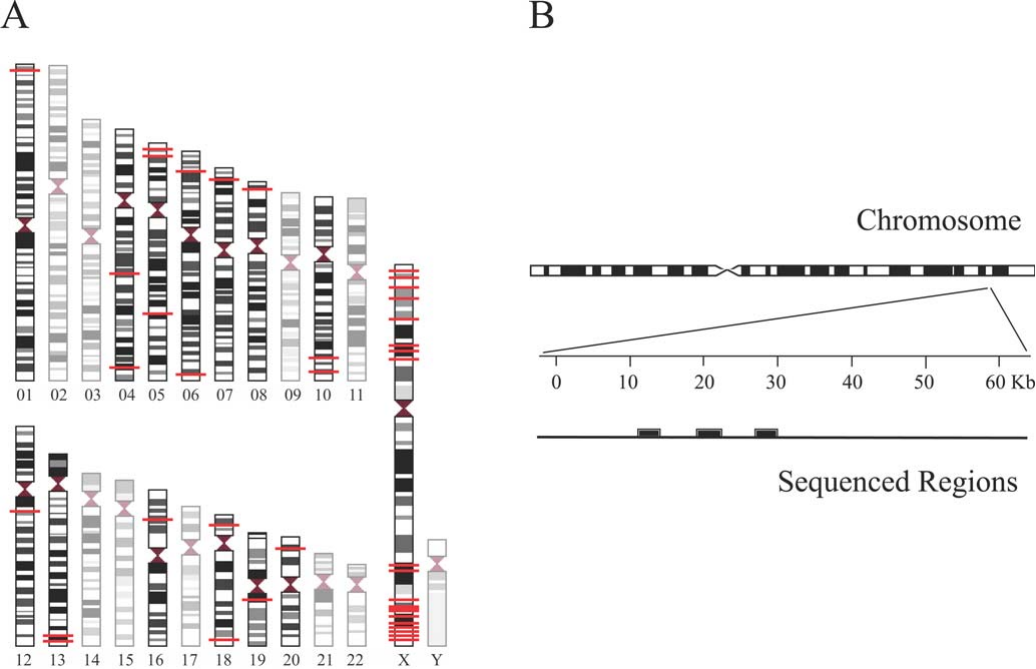
Loci under study. (A) Approximate chromosomal positions of 20 autosomal and 20 X-linked loci (red horizontal line). Each region encompasses ∼20 kb of single-copy non-coding (i.e., putatively non-functional) DNA in regions of medium or high recombination (r≥0.9 cM/Mb). (B) Sequencing strategy. Within each region, ∼4–6 Kb of sequence data were gathered from 3 or 4 discrete subsections (filled blocks) that spanned most of the distance of each region (see [14] for details).

In this paper, we analyze DNA sequence data that were collected by Wall et al. [14] for the purpose of testing models of human demographic history. In particular, we analyze data from the X chromosome and autosomes to examine the role that sex-specific processes have played in shaping genomic patterns of variability. We consider several alternative models that could lead to a skew in the ratio of X chromosome to autosomal diversity. Our sequence database includes 40 intergenic regions (20 on the X chromosome and 20 on the autosomes), each of which encompasses ∼20 kb of DNA (Figure 1). The sequenced regions were chosen from intergenic/non-coding (i.e., putatively non-functional) regions of medium or high recombination (r≥0.9 cM / Mb) to minimize any potential confounding effects of natural selection (see [14] for details). These data are also well-suited for testing the role of demographic processes in influencing patterns of diversity because all sites are resequenced in each individual, and multiple diverse human populations are represented in our survey (i.e., Biaka from Central African Republic, Mandenka from Senegal, San from Namibia, French Basque, Han Chinese and Melanesians from Papua New Guinea). We also utilize the recently available orangutan genome to obtain more accurate estimates of the underlying mutation rate for each of the regions studied.

## Results

**Figure 2:**
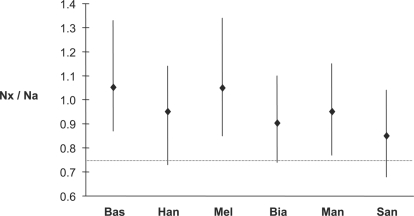
Ratio of effective population sizes for the X chromosome (*N_x_*) and autosomes (*N_a_*) for each population. The diamonds represent the point estimate, while the vertical bar shows the estimated 95% confidence interval. The dotted line represents the expected ratio (0.75) under a neutral model with breeding sex ratio of 1. Three letter population codes are as follows: Melanesians (Mel), Basque (Bas), Han Chinese (Han), Mandenka (Man), Biaka (Bia), San (San).

We analyze a total of ∼210 kb of DNA sequence representing 40 loci from the X chromosome and autosomes from each of 90 humans and three great apes, or a total of ∼18.9 Mb [14]. Table 1 provides basic summary statistics for nucleotide diversity in six human populations, as well as the ratio of diversity to human-orangutan sequence divergence. We also use levels of divergence between humans and orangutan (see Methods) to estimate mutation rates for each region (Table S1), and then estimate relative effective population sizes of the X chromosome and autosomes (*N_x_* / *N_a_*) based on observed levels of diversity (*θ_W_*) [14]. We find that this ratio is higher than expected in all six populations, ranging from 0.85 in the San to 1.08 in the Basque (Figure 2). When we use levels of divergence between humans and chimpanzees to estimate mutation rates for the autosomal and X-linked regions, we obtain similar results. For instance, X/A diversity ratios (e.g., π/D in Table 1) using chimpanzee and orangutan divergence are highly correlated for the six human populations (r^2^ = 0.95, P = 0.001) (data not shown). We also obtain similar π/D values when we subsample the human dataset to standardize the number of autosomes and X chromosomes (Table S2).

**Table 1:** Summaries of nucleotide diversity^a^ and divergence.

Population	Sample Size^b^	Segregating Sites	θ̅ (%)	π̅ (%)	D̅ (%)^c^	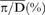	Tajima's D̅
Autosomes							
Mandenka	28.2	536	0.124	0.119	3.429	0.035	−0.140
Biaka	28.0	574	0.133	0.121	3.429	0.036	−0.343
San	19.5	500	0.133	0.125	3.421	0.037	−0.242
Han	32.0	352	0.079	0.080	3.425	0.023	0.045
Basque	32.0	336	0.076	0.086	3.426	0.025	0.509
Melanesians	18.0	281	0.073	0.078	3.426	0.022	0.327
X chromosome							
Mandenka	16.1	281	0.090	0.099	2.579	0.038	0.311
Biaka	14.0	281	0.093	0.095	2.586	0.036	0.093
San	9.0	220	0.083	0.085	2.584	0.033	0.147
Han	16.0	174	0.055	0.058	2.592	0.022	0.089
Basque	16.0	200	0.064	0.071	2.592	0.029	0.535
Melanesians	15.0	183	0.059	0.066	2.588	0.026	0.524

a Mean nucleotide diversity for 20 autosomal and 20 X-linked loci.

b Mean number of alleles sequenced per locus per population.

c D = human-orangutan sequence divergence.

Note—slight differences with values in [14] are due to alignment with different outgroup.

To test whether the observed ratios are significantly different from 0.75, we employ a maximum-likelihood method to estimate confidence intervals. Our method uses a population genetic model (i.e., the coalescent) to account for the inherent uncertainty in estimating diversity and divergence rates from sequence data. Figure 2 shows 95% confidence intervals for *N_x_* / *N_a_*. For three out of six populations (Basque, Melanesians and Mandenka), the 95% confidence intervals for the ratio of X-linked and autosomal effective population sizes does not include 0.75 (p = 0.001, 0.005 and 0.030 for the Basque, Melanesians and Mandenka, respectively). One interpretation of these results is that there is strong evidence for an unequal female and male *N_e_* in at least three of our six populations, with estimates of the breeding sex ratio (i.e., the effective size of females to males) ranging from 2.1 in the San to 12.5 in the Basque. If the observed differences in nucleotide variability on the X chromosome and autosomes are caused by long-term (demographic) processes, then the estimates of *N_x_* / *N_a_* presented in Figure 2 will be highly correlated due to shared population history. When we use the intersection of all six confidence intervals (0.87–1.02) to estimate the range of *N_x_* / *N_a_* values that are consistent with the data from all six populations, we estimate the range of the breeding sex ratio to be 2.4–8.7. We also note that even with a conservative Bonferroni correction, a 1∶1 breeding sex ratio is rejected in two out of six populations.

We also employ a separate method for estimating the breeding sex ratio in each population that does not allow for intra-locus recombination but does permit independent mutation rates across loci (see Methods). This method produces similar results to those described above, with estimates of the ratio of female to male effective population size ranging from 1.8 in the San to 14.0 in the Basque (Table S3). We interpret this as additional evidence that the unusual patterns observed in our data are real and require explanation.

## Discussion

Our findings of high levels of diversity on the X chromosome relative to the autosomes are in marked contrast to results of previous studies in a wide range of species including humans [15],[16], house mice [17], flycatcher [18], chicken [19], and Drosophila [12],[20] (but see [21]). Indeed, many evolutionary models, such as recent population bottlenecks [9],[10] and recurrent selective sweeps [11] are expected to *reduce* the relative levels of X-linked diversity, contrary to what we find.

Could our results be due to sequencing error? Since most of the samples used for sequencing came from males, the X chromosome data are essentially haploid while autosomal data are necessarily diploid. In principle, this could lead to a systematic bias in estimates of genetic diversity across different chromosomes. In particular, if diploid sequencing tends to miss rare variants, we might expect the estimates of autosomal diversity to be too low, leading to overestimates of *N_x_* / *N_a_*. Given that the levels of diversity for our autosomal loci [14] are higher than those found in other large-scale studies of human sequence data (e.g., [22]–[24]), we find this possibility to be highly unlikely. Moreover, there is no evidence that we have preferentially missed rare variants on the autosomes as mean Tajima's D values for our autosomal loci are comparable to those in other studies of non-genic regions [24] and more negative than those for our X-linked loci in five out of the six populations in Table 1.

The only other multi-locus study we are aware of that allows for a similar comparison of X versus autosomal diversity in thoroughly sampled, non-admixed human populations is the NIEHS SNPs study [25]. Since this study focuses on genic regions, it is not clear whether analyses of their data are directly comparable to the results described here. For example, genic regions in non-African populations of *Drosophila melanogaster* show reduced X-linked versus autosomal nucleotide diversity while intergenic regions do not (*i.e.*, X-linked and autosomal diversity levels are similar) [21]. Nonetheless, application of our methods to genes in the NIEHS study with recombination rates similar to those for our intergenic regions (i.e., r>0.9 cM/Mb) yields estimates of *N_x_* / *N_a_* ranging from 0.87 in the Yoruba to 1.08 in the CEPH (results not shown), similar to the point estimates shown in Figure 2. One interpretation of these results is that the long-term male effective population size is substantially smaller than the long-term female effective population size [7],[26]; however, other evolutionary processes may account for our observations. In the following sections we discuss four alternative models to explain the higher observed levels of X-linked to autosomal diversity than expected under neutral models.

### Background Selection

While directional selection on recessive beneficial mutations is expected to lead to more frequent hitchhiking and lower diversity on the X chromosome compared with the autosomes, linked negative selection on the X chromosome and autosomes (background selection) predicts the opposite pattern [11],[27]. Because recessive deleterious mutants are maintained at lower frequency and removed from populations more quickly on X chromosomes than on autosomes, neutral alleles on X chromosomes are less likely to be linked to a deleterious mutant compared with neutral alleles on autosomes. Thus, all else being equal, background selection should leave X chromosomes more polymorphic than autosomes at linked, neutral sites after correcting for expected differences in population size between X chromosomes and autosomes [12]. Because the effects of background selection are expected to be stronger (i.e., reduce local *N_e_*) in chromosomal regions with lower rates of recombination, we did not *a priori* believe that background selection would be a significant factor because our experimental design focuses on intergenic DNA in regions of moderate to high recombination [14]. To further explore the potential effects of background selection we assume an average number of deleterious mutations per generation of 4 [28] and use equation 15 in Hudson and Kaplan [29] to estimate the ratio of observed to expected polymorphism. We find this ratio to be 0.934, which suggests that background selection is unlikely to reduce autosomal diversity by more than 6.6% relative to X-linked diversity. We note that this estimate is conservative in that it ignores the effects of background selection on the X chromosome [30],[31]. Thus, it seems unlikely that background selection alone can explain our results. We also point out that alternative selection-based models involving the greater accumulation of sex-antagonistic polymorphisms on the sex chromosomes [32] may be viable.

### Demographic Processes Affecting the Entire Population

Historical changes in population size (such as founder effects and bottlenecks) also might have differential effects on loci with different modes of inheritance [9],[10],[33]. Using a simulation approach, we test three plausible models of recent demographic history that incorporate a recent population bottleneck and/or recent population growth. For example, we test a model incorporating 100-fold exponential growth from a constant effective population size of 10^4^, a bottleneck model with a 100-fold reduction in size followed by instantaneous recovery to an ancestral effective size of 10^4^, and a model incorporating the aforementioned bottleneck followed by 100-fold exponential growth (see Methods for details). For all parameters tested, the effects on expected relative levels of diversity are minor and in the direction towards reduced X-linked polymorphism (Table 2).

**Table 2:** Effect of demographic models on X versus autosome diversity. See Methods for details.

Time of growth (Kya)	Time of Bottleneck (Kya)	*θ̅* *_x_*/*θ̅* *_a_*
10	—	0.738
15	—	0.732
20	—	0.737
—	10	0.744
—	20	0.740
—	30	0.737
—	40	0.726
10	20	0.730
15	20	0.726
10	40	0.732
20	40	0.724

Recently, Pool and Nielsen [16] used an analytical approach to examine the effect of changing population sizes on the expected coalescence time for a pair of sequences with different effective population sizes. They showed that population size reductions can lead to particularly low X-linked diversity, whereas population growth can elevate X-linked relative to autosomal diversity. We employ Pool and Nielsen's [16] model (which is similar to the bottleneck model described above), substituting parameters that are reasonable for human demographic history (see Text S1 for details). When we examine the effects of such a bottleneck over a range of times in the past, we do not find that the expected X/A diversity ratio shifts much above 0.75 (Figure S1A). When we search for combinations of parameters that yield X/A diversity ratios and levels of nucleotide diversity similar to those that we observe, we find that ancient bottlenecks (i.e., older than ∼100 kya) coupled with population growth, can indeed produce expected X/A diversity ratios as high as 0.85 (Figure S1B). However, computer simulations using these same bottleneck and growth parameters yield summaries of the site frequency spectrum that are inconsistent with those that we observe; i.e., Tajima's D values that are much more negative than those in Table 1 for both the X chromosome and autosomes (−1.55 and −1.91, respectively).

### Sex-Biased Forces

There are a number of sex-biased evolutionary forces acting within human populations that are known to have differential effects on loci with different modes of inheritance. A demographic process that may lead to a skew in X-linked versus autosomal diversity is differential migration rates for males and females in a structured population. To explore the effects of sex-biased migration on ratios of X/A diversity, we simulate a two-deme island model with different rates of male and female migration. First, we simulate a symmetric model with only a single sex (females) migrating. We assume effective population sizes and migration rates that produce F_ST_ values that are similar to those observed in human populations (i.e., autosomal F_ST_ ∼0.12; [14]). Because females are exchanging demes at the same rate, it is not surprising that this model yields X/A diversity ratios that are close to those expected under panmixia (i.e., 0.75) (Table S4). Second, we simulate a model in which one deme sends out females and the other deme sends out males at the same rate. The results indicate that when the X-linked diversity exceeds the value expected under panmixia in one deme, the other deme always shows a deficit of X-linked diversity (Table S4). If we assume that the six populations that we sampled here evolve independently according to this two-deme model, the probability of observing excess polymorphism on the X chromosome for all six populations would be at most 1/64 (P<0.016). These results are consistent with Laporte and Charlesworth's [34] simulations showing that sex-biased migration only weakly skews levels of X/A diversity unless populations are strongly subdivided. Therefore, we believe that population structure is unlikely to generate a bias towards increased diversity on the X chromosome in all populations, but could contribute to differential bias among populations.

A higher variance in male reproductive success over that in females due to sexual selection is also expected to inflate the ratio of X-linked to autosomal polymorphism. In populations with age structure, an additional contribution to the variance in net reproductive success can be caused by the stochastic nature of survival during the reproductive phase and by differences in fertility among individuals in different age classes [26]. However, demographic factors of this kind (e.g., lower male survival during adult life or delayed male versus female age of maturity) are unlikely to have a major effect on the relative effective population sizes of X-linked and autosomal loci [26]. In contrast, an excess variance of male reproductive success over Poisson expectations can have large effects: With an extremely high variance in male fertility relative to female fertility, the ratio *N_x_* / *N_A_* approaches 1.125 [7],[26],[34].

### Conclusion

A number of evolutionary forces may be responsible for increasing the effective population size of X-linked versus autosomal loci. Under reasonable parameters for human populations, our results suggest that background selection, changes in population size, and sex-specific migration in a structured population may each have a minor effect in increasing the ratio of X-linked to autosomal polymorphism over that expected under neutral models. While it is possible that multiple processes acting together might lead to a major effect (i.e., on the order of what is observed here), we hypothesize that a higher variance in male versus female reproductive success can by itself explain most of the observed increase in effective population size of the X chromosome. The human mating system is considered to be moderately polygynous, based on both surveys of world populations [35],[36] and on characteristics of human reproductive physiology [37]–[39]. The practice of polygyny, in both the traditional sense and via ‘effective polygyny’ (whereby males tend to father children with more females than females do with males—a common practice in many contemporary western cultures [40]), would tend to increase the variance in reproductive success among males. In other words, when more men than women in any generation fail to have any children, and more men than women have very large numbers of children, autosomal *N_e_* is reduced relative to that of the X chromosome. While polygyny may be the most important factor influencing the ratio of X-linked to autosomal diversity, we point out that this process by itself is unlikely to account for all the patterns of nucleotide polymorphism observed here (e.g., the frequency spectrum as summarized by Tajima's D in Table 1). Future theoretical work examining the joint effects of multiple demographic processes (e.g., sex-biased bottlenecks in which populations are founded by more females than males (e.g., [21]) and experimental research (e.g., aimed at refining estimates of the ratio of X-linked to autosomal neutral polymorphism in additional populations) will increase our understanding of how the different forces of evolution influence variation on the autosomes and X chromosome.

## Methods

### Population Samples and Genomic Regions Sequenced

The DNA samples used in this study come from the CEPH Human Genome Diversity Panel [41], the YCC collection [42], and established collections in the Hammer lab (see [14] for details). The regions used for sequencing were selected to minimize any potential confounding effects of natural selection. Specifically, we identified 40 different intergenic (i.e., putatively non-functional) regions of ∼20 Kb in length with to medium to high recombination (r≥0.9 cM/Mb) [43]. While the genome-wide average recombination rate (mean±SE) for autosomes and the X chromosome are ∼1.29±0.018 cM/Mb and ∼1.25±0.091 cM/Mb, respectively [43], the average recombination rate for our autosomal and X-linked loci are 2.18±0.16 and 2.29±0.23, respectively. Each region was at least 50 Kb (100 Kb for the autosomes) away from the nearest gene; within each region, we gathered ∼4–6 Kb of sequence data from 3 or 4 discrete subsections that spanned most of the distance of each region (*locus trio*). For more details on the sequenced regions and the sequencing strategy, see Wall et al. [14]. See Table S5 for the number of alleles sequenced at each locus.

### Estimation of Effective Population Sizes of the X Chromosome and Autosomes (N_X_ / N_A_)

We used a maximum-likelihood framework for estimating the effective population size for the X chromosome (denoted by *N_x_*) and for the autosomes (*N_a_*). We did this separately for each of the six study populations. We tabulated the number of segregating sites and the number of fixed differences (between human and orangutan) for each locus, and then used coalescent simulations [44] to estimate the probability of these observations as a function of the population size and the mutation rate. Similar results were obtained when the chimpanzee was used as an outgroup.

We assumed an average generation time of *g* = 25 years for all human generations since the human most recent common ancestor (MRCA) and an average generation time of *g* = 20 years for all generations between the human MRCA and the orangutan sequence. We fixed the human–orangutan split time at 15 million years ago and assumed an ancestral human–orangutan population size of 40,000 for the autosomes and 30,000 for the X chromosome. The mutation rate was assumed to be constant per base pair, but different for the X (*μ_X_*) and the autosomes (*μ_A_*).

For a specific population, let *S_Ai_* denote the number of segregating sites in the *i*-th autosomal locus and let *S_Xj_* denote the number of segregating sites in the *j*-th X-linked locus. Similarly, let *D_Ai_* and *D_Xj_* denote the number of fixed differences between human and orangutan at the *i*-th autosomal and the *j*-th X-linked locus respectively. We correct *D_Ai_* and *D_Xj_* for multiple hits using the Jukes-Cantor model [45]. Then, the likelihood we are interested in is
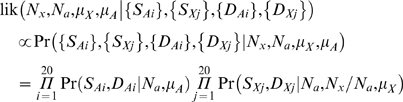
(1)Our basic strategy is to consider a grid of *μ_A_*, *μ_X_*, *N_A_*, and (*N_X_* / *N_A_*) values and to use Monte Carlo coalescent simulations to estimate (1) for each grid point. In particular, *μ_A_* and *μ_X_* are incremented in units of 0.1×10^−8^/bp per generation, *N_a_* is incremented in units of 500, and (*N_x_* / *N_a_*) is incremented in units of 0.05. At each locus we generate 10^5^ ancestral recombination graphs (ARGs) of *n* = 9–34 human sequences (corresponding to the sample size for the actual data) and one orangutan sequence, reproducing both the actual lengths sequenced and the gaps between the sequenced segments. These ARGs have a recombination rate that is constant per base pair, with the rate estimated from the deCODE map [43], assuming an effective population size of 12,500. Next, we tabulated the total branch lengths of branches that would lead to segregating sites or fixed differences. For any particular set of parameter values {*μ_A_*, *μ_X_*, *N_a_*, and (*N_x_* / *N_a_*)}, it is straightforward to calculate the expected number of segregating sites and fixed differences under the infinite-sites model. Denote these by *ES* and *ED* respectively. Then, the probabilities in (1) follow from the Poisson distribution, and
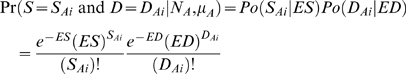
for the autosomal loci or
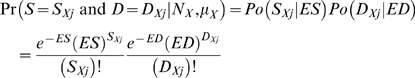
for the X-linked loci.

Note that the same set of simulations is used to estimate probabilities for a locus over all grid points simultaneously. This added computational efficiency comes at the cost of assuming ρ / bp (for the ARGs) is the same across all different values of *N_a_* and *N_x_*. Simulations assume a constant population size and no population structure for each human population. The results are somewhat robust to specific demographic assumptions (see below).

### Estimating the Ratio of Female to Male Effective Population Size (Breeding Sex Ratio)

Denote the female effective population size by *N_f_* and the male effective population size by *N_m_*. We use two separate approaches for estimating the breeding sex ratio *α* = *N_f_* / *N_m_*. First, we use a method of moments approach to obtain point estimates of *α*. Define *ß* = *N_x_* / *N_a_*. From standard population genetics [8],
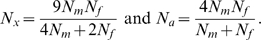
Substituting and rearranging terms leads to
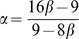
We then substitute the point estimates for *ß* obtained above to generate point estimates for *α*.

The second method to estimate the breeding sex ratio is a likelihood-based approach similar to the method for estimating *N_x_* / *N_a_* described above. As before, we use maximum-likelihood to obtain a point estimate (of *α*) and likelihood-ratio tests to estimate 95% confidence intervals, separately for each of the six populations. In this approach, we assume no recombination within loci, free recombination between loci, and no variation in coalescence times of lines in the ancestral human-orangutan population across the genome. Unlike the previous method, we assume that the mutation rates are not constant across loci. Denote the mutation rates at the i-th autosomal locus and the j-th X-linked locus by *μ_Ai_* and *μ_Xj_*, respectively. Using the same notation as before, the desired likelihood is
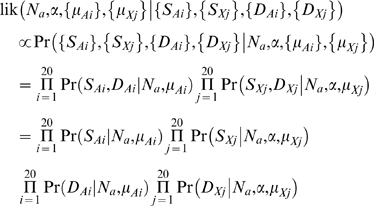
Since each locus is independent, we can simply maximize the likelihood over *μ*, *N_a_* and *α* separately for each locus.

For the divergence terms the probability is Poisson distributed:
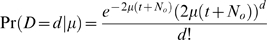
where *t* is the number of generations since the human-orangutan split and *N_o_* is the effective population size of the ancestral human-orangutan population for the locus in question. For the polymorphism terms, we utilize an exact expression that is available for the standard coalescent without recombination [46]:
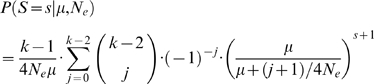
where *N_e_* is the effective population size of the locus and *k* is the sample size. Note that no simulations are necessary for calculating likelihoods. Point estimates for *α* (as well as 95% CI) are shown in Table S1. Results on the performance and robustness of the various estimation methods used are described in Text S1.

### Sensitivity of Estimates of *N_x_* / *N_a_* to Demographic Assumptions

To test whether alternative demographic models might influence the observed ratio *N_x_* / *N_a_*, we considered simple models that incorporated a population bottleneck and/or recent population growth. These simulations assumed *N_x_* = 7,500, *N_a_* = 10,000, *g* = 25 years, *θ* = ρ = 0.001 / bp in the ancestral population, *n* = 32 for the autosomes and *n* = 16 for the X chromosome. Our growth only model assumed that a population of constant size began growing exponentially at various times in the past (i.e, 10, 15, and 20 kya), expanding to a size 100-fold larger than the ancestral population. Our bottleneck only model assumed that an ancestral population underwent a 100-fold decrease in size at various times in the past (i.e., 10, 20, 30, and 40 kya) before instantaneously recovering its original size. In all cases the bottleneck lasted for 40 generations. We also considered a modification of the bottleneck model where the population grows exponentially at various times (i.e., 10, 15, and 20 kya) after recovering from the bottleneck described above (for cases where the onset of the bottleneck was 20 and 40 kya). For each parameter combination, we simulated 10^4^ replicates of a 5 Kb region, and tabulated *θ̅* *_x_*/*θ̅* *_a_*.

We then considered simple two-deme island models to test the effects of sex-biased migration rates on *θ̅* *_x_*/*θ̅* *_a_*. Each population experiences a per-generation migration rate of 3–9×10^−5^ and an effective population size of 10^4^. We test symmetric migration models in which females and males migrate equally between demes and in which only females or only males migrate between demes, as well as asymmetric models in which females and males migrate in opposite directions between demes. We performed 10,000 simulations for each model (Table S2).

## Supporting Information

Figure S1 Predicted ratio of X chromosome to autosome diversity for two bottleneck models.
(0.28 MB DOC)

Table S1 Mutation rates at 40 loci assuming a 15-million year human-orangutan divergence time.
(0.04 MB DOC)

Table S2 Polymorphism and divergence for autosomal and X-linked loci.
(0.04 MB DOC)

Table S3 Breeding sex ratio.
(0.03 MB DOC)

Table S4 Migration rates and simulation results for two-deme migration model.
(0.04 MB DOC)

Table S5 Sample sizes (number of alleles sequenced) for each locus.
(0.06 MB DOC)

Text S1 Supplementary material.
(0.04 MB DOC)
