# Supplementary material for: Sex-Biased Evolutionary Forces Shape Genomic Patterns of Human Diversity
Source: PLoS Genet. 2008 Sep 26;4(9):e1000202. doi: 10.1371/journal.pgen.1000202 (PMC2538571; doi:10.1371/journal.pgen.1000202)
Supplement: Figure S1 — Predicted ratio of X chromosome to autosome diversity for two bottleneck models. (0.28 MB DOC) [file pgen.1000202.s001.doc]

Figure S1. Predicted ratio of X chromosome to autosome diversity for two bottleneck models (see Pool and Nielsen 2007). (A) Recent bottleneck. A population with initial N = 10,000 experiences a reduction in size for 100 generations followed by instantaneous growth to original size. (B) Ancient bottleneck and growth. A population with initial N = 5,750 experiences a reduction in size for 138 generations followed by instantaneous growth to its original size, and then 40-fold growth to current size. *1/f* is the strength of the bottleneck measured from current size, and *g* represents the time since the end of the bottleneck in generations.

A.

B.


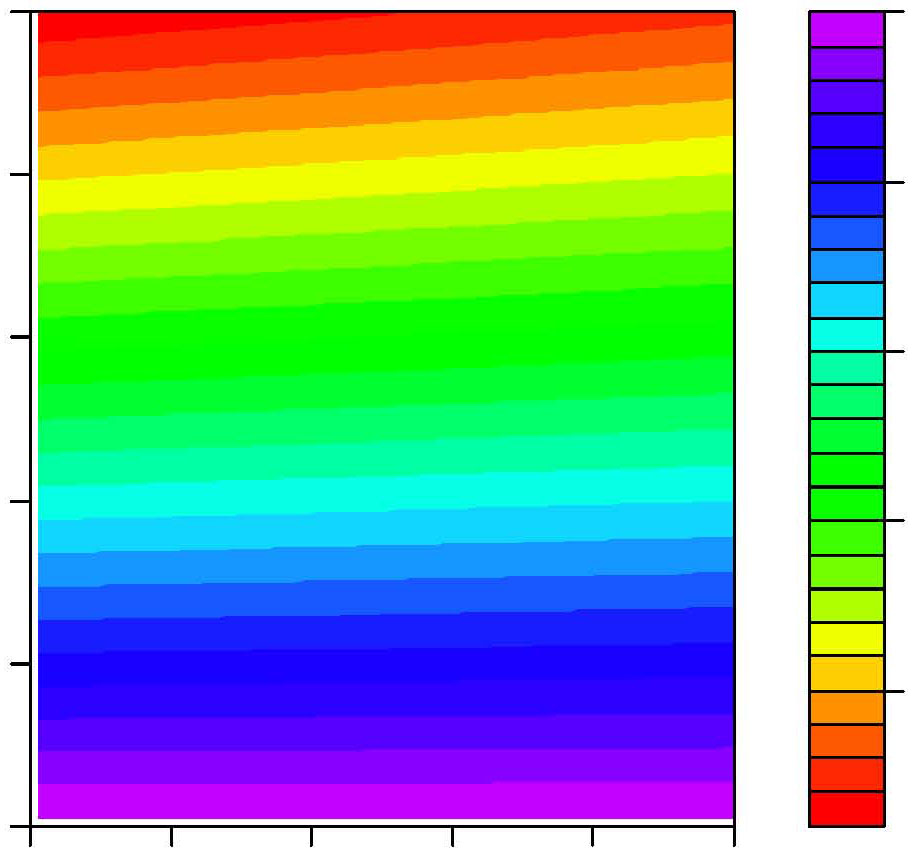


0 100 200 300 400 500

0

20

40

60

80

100

g

1/f

0.71

0.72

0.73

0.74

0.75

X/A ratio


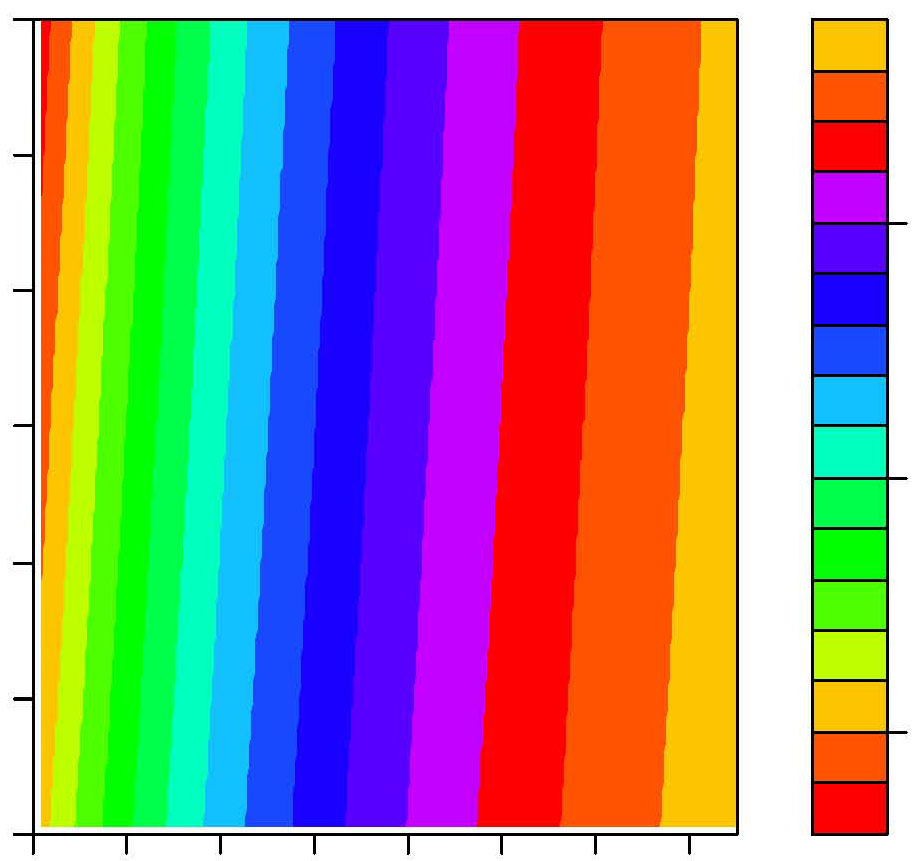


0 2000 6000 10000 14000

0

100

150

200

250

300

1/f

0.75

0.80

0.85

X/A ratio

50

g
