# Supplementary material for: Sex-Biased Evolutionary Forces Shape Genomic Patterns of Human Diversity
Source: PLoS Genet. 2008 Sep 26;4(9):e1000202. doi: 10.1371/journal.pgen.1000202 (PMC2538571; doi:10.1371/journal.pgen.1000202)
Supplement: Table S1 — Mutation rates at 40 loci assuming a 15-million year human-orangutan divergence time. (0.04 MB DOC) [file pgen.1000202.s002.doc]

Table S1. Mutation rates at 40 loci assuming a 15-million year human-orangutan divergence time.

|  | µ (bp/year) |
| --- | --- |
| 10qMB119 | 8.497E-10 |
| 10qMB128 | 1.277E-09 |
| 12qMB46 | 1.103E-09 |
| 13qMB107 | 1.001E-09 |
| 13qMB108 | 1.055E-09 |
| 16pMB17 | 9.249E-10 |
| 18pMB7 | 1.065E-09 |
| 18qMB73 | 1.268E-09 |
| 19qMB35 | 1.219E-09 |
| 1pMB4 | 1.261E-09 |
| 20pMB7 | 9.470E-10 |
| 4qMB105 | 1.254E-09 |
| 4qMB181 | 1.056E-09 |
| 5pMB10 | 1.278E-09 |
| 5pMB4 | 1.359E-09 |
| 5qMB128 | 7.994E-10 |
| 6pMB14 | 9.859E-10 |
| 6qMB164 | 1.248E-09 |
| 7pMB8 | 1.128E-09 |
| 8pMB5 | 1.790E-09 |
| *mean* | 1.14E-09 |
|  |  |
| XpMB13 | 6.291E-10 |
| XpMB22 | 8.800E-10 |
| XpMB3 | 9.647E-10 |
| XpMB33 | 7.369E-10 |
| XpMB35 | 9.212E-10 |
| XpMB39 | 9.824E-10 |
| XpMB6 | 8.784E-10 |
| XpMB9 | 8.237E-10 |
| XqMB120 | 8.925E-10 |
| XqMB124 | 6.963E-10 |
| XqMB136 | 8.024E-10 |
| XqMB139 | 6.734E-10 |
| XqMB140 | 9.768E-10 |
| XqMB141 | 9.003E-10 |
| XqMB143 | 8.537E-10 |
| XqMB145 | 1.184E-09 |
| XqMB146 | 1.047E-09 |
| XqMB148 | 7.298E-10 |
| XqMB149 | 7.136E-10 |
| XqMB150 | 9.184E-10 |
| *mean* | 8.60E-10 |
