# Supplementary material for: Sex-Biased Evolutionary Forces Shape Genomic Patterns of Human Diversity
Source: PLoS Genet. 2008 Sep 26;4(9):e1000202. doi: 10.1371/journal.pgen.1000202 (PMC2538571; doi:10.1371/journal.pgen.1000202)
Supplement: Table S2 — Polymorphism and divergence for autosomal and X-linked loci. (0.04 MB DOC) [file pgen.1000202.s003.doc]

| Table S2. Polymorphism^a^ and divergence for autosomal and X-linked loci | | | | | |
| --- | --- | --- | --- | --- | --- |
|  | Population | Sample Size | Segregating Sites | π (%) | π /D^b^ (%) |
| Autosomes |  |  |  |  |  |
|  | Mandenka | 14 | 374 | 0.122 | 0.036 |
|  | Biaka | 14 | 383 | 0.122 | 0.036 |
|  | San | 9 | 293 | 0.120 | 0.035 |
|  | Han | 16 | 242 | 0.081 | 0.024 |
|  | Basque | 16 | 250 | 0.090 | 0.027 |
|  | Melanesians | 14 | 243 | 0.081 | 0.025 |
| X chromosome | |  |  |  |  |
|  | Mandenka | 14 | 220 | 0.098 | 0.038 |
|  | Biaka | 14 | 219 | 0.095 | 0.036 |
|  | San | 9 | 167 | 0.085 | 0.033 |
|  | Han | 16 | 133 | 0.058 | 0.022 |
|  | Basque | 16 | 162 | 0.071 | 0.027 |
|  | Melanesians | 14 | 128 | 0.067 | 0.025 |
| ^a^ After subsampling to standardize the number of autosomes and X chromosomes | | | | | |
| ^b^ Human-Orangutan divergence | | |  |  |  |
