# Supplementary material for: Sex-Biased Evolutionary Forces Shape Genomic Patterns of Human Diversity
Source: PLoS Genet. 2008 Sep 26;4(9):e1000202. doi: 10.1371/journal.pgen.1000202 (PMC2538571; doi:10.1371/journal.pgen.1000202)
Supplement: Table S3 — Breeding sex ratio. (0.03 MB DOC) [file pgen.1000202.s004.doc]

| Table S3. Breeding Sex Ratio^a^ | | |
| --- | --- | --- |
| Population | MLE | 95%CI |
| Basque | 14.0 | (1.8 - ∞) |
| Han | 2.5 | (0.4 - ∞) |
| Melanesians | 6.0 | (1.0 - ∞) |
| Biaka | 2.9 | (0.6 - ∞) |
| Mandenka | 4.3 | (0.8 - ∞) |
| San | 1.8 | (0.2 - ∞) |
| ^a^ Based on human - orangutan divergence | | |
