# Supplementary material for: Sex-Biased Evolutionary Forces Shape Genomic Patterns of Human Diversity
Source: PLoS Genet. 2008 Sep 26;4(9):e1000202. doi: 10.1371/journal.pgen.1000202 (PMC2538571; doi:10.1371/journal.pgen.1000202)
Supplement: Table S4 — Migration rates and simulation results for two-deme migration model. (0.04 MB DOC) [file pgen.1000202.s005.doc]

| Table S4. Migration rates^a^ and simulation results^b^ for two-deme migration model. | | | | | | |
| --- | --- | --- | --- | --- | --- | --- |
|  | m*_12_*^c^ | m*_12_* | m*_21_* | m*_21_* | X/A^d^ | X/A^e^ |
| Model | males | females | males | females | deme 1 | deme 2 |
| Asymmetric |  | 0.45 | 0.45 |  | 0.750 | 0.650 |
| " |  | 0.15 | 0.15 |  | 0.800 | 0.640 |
| Symmetric (Females) |  | 0.45 |  | 0.45 | 0.750 | e |
| " |  | 0.15 |  | 0.15 | 0.754 | e |
| Symmetric (Males) | 0.45 |  | 0.45 |  | 0.744 | e |
| " | 0.15 |  | 0.15 |  | 0.754 | e |
| Symmetric (both sexes) | 0.45 | 0.45 | 0.45 | 0.45 | 0.758 | e |
| " | 0.15 | 0.15 | 0.15 | 0.15 | 0.742 | e |
| ^a^ Number of migrants per generation | |  |  |  |  |  |
| ^b^ Based on an effective population size of 5,000 for each sex. | | | |  |  |  |
| ^c^ Sub-index *xy* indicates migration to deme *x* from deme *y* | | | |  |  |  |
| ^d^ Mean ratio of X-linked to autosomal diversity in 10,000 simulations | | | | |  |  |
| ^e^ Estimated expected value equal to value in deme 1 | | | |  |  |  |
