# Supplementary material for: Sex-Biased Evolutionary Forces Shape Genomic Patterns of Human Diversity
Source: PLoS Genet. 2008 Sep 26;4(9):e1000202. doi: 10.1371/journal.pgen.1000202 (PMC2538571; doi:10.1371/journal.pgen.1000202)
Supplement: Table S5 — Sample sizes (number of alleles sequenced) for each locus. (0.06 MB DOC) [file pgen.1000202.s006.doc]

|  | Locus | | | | | | | | | | | | | | | | | | | |  |
| --- | --- | --- | --- | --- | --- | --- | --- | --- | --- | --- | --- | --- | --- | --- | --- | --- | --- | --- | --- | --- | --- |
|  | 10q MB119 | 10q MB128 | 12q MB46 | 13q MB107 | 13q MB108 | 16p MB17 | 18p MB7 | 18q MB73 | 19q MB35 | 1p MB4 | 20p MB7 | 4q MB105 | 4q MB181 | 5p MB10 | 5p MB4 | 5q MB128 | 6p MB14 | 6q MB164 | 7p MB8 | 8p MB5 | mean |
| Mandenka | 28 | 28 | 30 | 28 | 28 | 28 | 28 | 28 | 28 | 28 | 28 | 28 | 28 | 30 | 28 | 28 | 28 | 28 | 28 | 28 | 28.2 |
| Biaka | 28 | 28 | 28 | 28 | 28 | 28 | 28 | 28 | 28 | 28 | 28 | 28 | 28 | 28 | 28 | 28 | 28 | 28 | 28 | 28 | 28.0 |
| San | 28 | 18 | 18 | 18 | 18 | 28 | 18 | 18 | 18 | 18 | 18 | 28 | 18 | 18 | 18 | 18 | 18 | 18 | 18 | 18 | 19.5 |
| Han | 32 | 32 | 32 | 32 | 32 | 32 | 32 | 32 | 32 | 32 | 32 | 32 | 32 | 32 | 32 | 32 | 32 | 32 | 32 | 32 | 32.0 |
| Basque | 32 | 32 | 32 | 32 | 32 | 32 | 32 | 32 | 32 | 32 | 32 | 32 | 32 | 32 | 32 | 32 | 32 | 32 | 32 | 32 | 32.0 |
| Melanesians | 18 | 18 | 18 | 18 | 18 | 18 | 18 | 18 | 18 | 18 | 18 | 18 | 18 | 18 | 18 | 18 | 18 | 18 | 18 | 18 | 18.0 |
|  |  |  |  |  |  |  |  |  |  |  |  |  |  |  |  |  |  |  |  |  |  |
|  | Xp MB13 | Xp MB22 | Xp MB33 | Xp MB35 | Xp MB39 | Xp MB3 | Xp MB6 | Xp MB9 | Xq MB120 | Xq MB124 | Xq MB136 | Xq MB139 | Xq MB140 | Xq MB141 | Xq MB143 | Xq MB145 | Xq MB146 | Xq MB148 | Xq MB149 | Xq MB150 | Mean |
| Mandenka | 18 | 14 | 14 | 14 | 14 | 18 | 16 | 14 | 18 | 14 | 18 | 14 | 14 | 18 | 14 | 18 | 18 | 18 | 18 | 18 | 16.1 |
| Biaka | 14 | 14 | 14 | 14 | 14 | 14 | 14 | 14 | 14 | 14 | 14 | 14 | 14 | 14 | 14 | 14 | 14 | 14 | 14 | 14 | 14.0 |
| San | 9 | 9 | 9 | 9 | 9 | 9 | 9 | 9 | 9 | 9 | 9 | 9 | 9 | 9 | 9 | 9 | 9 | 9 | 9 | 9 | 9.0 |
| Han | 16 | 16 | 16 | 16 | 16 | 16 | 16 | 16 | 16 | 16 | 16 | 16 | 16 | 16 | 16 | 16 | 16 | 16 | 16 | 16 | 16.0 |
| Basque | 16 | 16 | 16 | 16 | 16 | 16 | 16 | 16 | 16 | 16 | 16 | 16 | 16 | 16 | 16 | 16 | 16 | 16 | 16 | 16 | 16.0 |
| Melanesians | 14 | 14 | 14 | 14 | 14 | 14 | 14 | 14 | 14 | 14 | 16 | 16 | 16 | 16 | 16 | 16 | 16 | 16 | 16 | 16 | 15.0 |

Table S5. Sample sizes (number of alleles sequenced) for each locus.
