# Supplementary material for: Sex-Biased Evolutionary Forces Shape Genomic Patterns of Human Diversity
Source: PLoS Genet. 2008 Sep 26;4(9):e1000202. doi: 10.1371/journal.pgen.1000202 (PMC2538571; doi:10.1371/journal.pgen.1000202)
Supplement: Text S1 — Supplementary material. (0.04 MB DOC) [file pgen.1000202.s007.doc]

# Text S1

Testing the likelihood-based methods for estimating N_x_ / N_a_

We ran coalescent simulations (Hudson 2002) to test the accuracy of our methods for estimating N_x_ / N_a_. Specifically, we simulated sequence data comparable to the actual data, then calculated a point estimate and a 95% confidence interval for N_x_ / N_a_ on the simulated data. These simulations assumed that N_x_ = 9,000, N_a_ = 12,000 and µ = 2.5 * 10^-8^ / bp (i.e., the “true” N_x_ / N_a_ is 0.75). Each replicate involved simulating 20 X-linked and 20 autosomal loci, and each locus consisted of 5 Kb of sequenced data from a region 17 Kb long. We simulated a sample size of n = 32 for the autosomal loci and n = 16 for the X-linked loci, as well as a single outgroup (orangutan) sequence.

We considered two different scenarios for recombination, each with the same average rate of r = 1.5 * 10^-8^ / bp. The first scenario assumed that the recombination rate was constant across sites. The second scenario modeled recombination hotspots, assuming hotspots of 1 Kb occurred with an average density of one hotspot per 20 Kb. We assumed that hotspots are non-overlapping and that the distance between hotspots followed a negative binomial distribution with mean 19 Kb. The relative rates of recombination in hotspot and non-hotspot regions were chosen so that 5% of the sequence contained 50% of the total recombination. We ran 100 replicates for each scenario. This took approximately one week of computing time on a standard Intel Xeon processor.

For each replicate, we calculated the point estimate and 95% confidence interval for N_x_ / N_a_. We then tabulated the mean and the standard deviation of the N_x_ / N_a_ estimates and determined what proportion of the time the true value of N_x_ / N_a_ (0.75) was contained in the estimated 95% confidence interval. The results of these simulations are summarized below. Both sets of simulations suggest that estimates of N_x_ / N_a_ are roughly unbiased, and there was no significant difference between the distributions of estimated N_x_ / N_a_ values under the two different recombination models. The coverage properties of the 95% confidence interval may be slightly non-conservative under the hotspot model, though we did not run enough trials to determine this definitively and the simulation results are consistent with null expectations.

|  | Mean estimate | SD of estimate | Coverage |
| --- | --- | --- | --- |
| Uniform rec. | 0.75 | 0.09 | 0.96 |
| Hotspots | 0.76 | 0.08 | 0.92 |

To evaluate the performance of our second method, we simulated paired datasets with recombination (r = 2.3925 * 10^-8^ Morgans/bp) and without recombination (Hudson 2002) for a sequence of about 5Kb within a region of about 19Kb . The datasets were generated under three different female to male mating ratios: 1:1, 4:1, 7:1, under the standard neutral model. Each dataset contained 20-X linked and 20 autosomal loci, with samples sizes of n=32 for the autosomal loci and n=16 for the X-linked loci, and including a single outgroup (orangutan) sequence. In all cases the actual mating ratio was within the range of accepted values. We conclude that the assumption of no recombination doesn't seem to affect the performance of the test.

# Exploring parameters for a bottleneck/growth model to explain the observed X/A ratio

We analyzed a bottleneck model with five parameters: current effective size, bottleneck size, pre-bottleneck size, time since the end of the bottleneck, and duration of the bottleneck. We explored a range of values for each of the parameters and, using exact expressions for the expected value of nucleotide diversity (Pool and Nielsen 2007) for the X chromosome and the autosomes, we found conditions that were consistent with a constant effective size for the autosomes of 9,900-12,100 and a ratio of expected X / A nucleotide diversity of 0.808-0.879. We considered the following ranges for a 5-parameter bottleneck model with constant size in each phase of the model:

current effective size: 10000 - 200000 individuals

ratio of bottleneck population size to current population size: 0.001 - 1

ratio of pre-bottleneck population size to current population size: 0.001 - 1

generations since the end of the bottleneck: 400 - 10000

duration of the bottleneck in generations :1 – 1000

For each parameter, we considered 20 values, totaling 3,200,000 evaluations of conditions. Of those conditions that met the specifications for long-term effective population size and the ratio of expected X / A nucleotide diversity, we simulated 1,000 datasets of 20 sequences corresponding to the autosomes using the program ms (Hudson 2002). We then generated distributions of averages of Tajima's D for each set of 20 sequences, and the 1,000 averages were sorted. The values for the percentiles were estimated from the sorted averages. In all cases the value for the 97.5 percentile was more negative than each of the observed values in our samples.
